# Effects of Switching from Anti-VEGF Treatment to Triamcinolone Acetonide in Eyes with Refractory Macular Edema Associated with Diabetic Retinopathy or Retinal Vein Occlusion

**DOI:** 10.1155/2020/4529850

**Published:** 2020-11-20

**Authors:** Tomoaki Tatsumi, Toshiyuki Oshitari, Takayuki Baba, Yoko Takatsuna, Shuichi Yamamoto

**Affiliations:** ^1^Department of Ophthalmology and Visual Science, Chiba University Graduate School of Medicine, 1-8-1, Inohana, Chuo-ku, Chiba City, Chiba 260-8670, Japan; ^2^Department of Ophthalmology, International University of Health and Welfare, 4-3, Kozunomori, Narita City, Chiba 286-8686, Japan; ^3^Department Ophthalmology, Chiba Rosai Hospital, 2-16, Tatsumidaihigashi, Ichihara City, Chiba 290-0003, Japan

## Abstract

**Purpose:**

To evaluate the efficacy of switching from intravitreal antivascular endothelial growth factor (VEGF) agents to triamcinolone acetonide (TA) in eyes with diabetic macular edema (DME) or with retinal vein occlusion-associated macular edema (RVO-ME) on the resolution of the macular edema (ME).

**Methods:**

The medical records of 11 eyes of 11 patients with DME and 9 eyes of 9 patients with RVO-ME whose MEs were refractory to anti-VEGF treatment were reviewed. The central retinal thickness (CRT), best-corrected visual acuity (BCVA), intraocular pressure (IOP), and the mean interval of the recurrences were measured during the anti-VEGF treatment and after switching to the TA injections.

**Results:**

Switching to TA injections significantly increased the mean interval for recurrences from 9.2 ± 2.7 weeks to 22.3 ± 12.9 weeks in eyes with DME (*P* = 0.006). In eyes with RVO-ME, the mean period of recurrence was 12.3 ± 5.6 weeks before and 11.6 ± 4.4 weeks after the switch (*P* = 0.44). The mean interval for recurrence was extended to more than 8 weeks in 7 of 11 eyes with DME, but none of the eyes with RVO-ME had a prolongation of more than 4 weeks. An elevation of the IOP was observed in 3 of the 20 eyes after the TA injection.

**Conclusions:**

These findings indicate that switching to TA injections can be a good option for DME eyes refractory to anti-VEGF injections but not for the RVO-ME eyes.

## 1. Introduction

Diabetic macular edema (DME) is the most common cause of reduced visual acuity in patients with nonproliferative diabetic retinopathy [1]. A meta-analysis found that 6.81% of 22,896 diabetic patients had DME [2]. Intravitreal injections of antivascular endothelial growth factor (anti-VEGF) agents such as bevacizumab, ranibizumab, and aflibercept have become the standard therapy for DME worldwide [3, 4].

A retinal vein occlusion (RVO) is the second most common cause of visual impairments after diabetic retinopathy [5, 6]. The most common cause of visual impairments in patients with branch retinal vein occlusion (BRVO) and chronic central retinal vein occlusion (CRVO) is macular edema (ME) [6–9]. In the treatment of the ME associated with retinal vein occlusion (RVO-ME), the efficacy of intravitreal injections of anti-VEGF agents has been demonstrated in many large-scale clinical trials [10–16].

Although anti-VEGF treatments have been successful in resolving the DME and RVO-ME, there is a high rate of recurrences [17]. Thus, multiple anti-VEGF injections are required to maintain the resolution of the ME.

In Japan, anti-VEGF treatments are relatively expensive, which puts an economic burden on the patients. The cost of anti-VEGF agents, which is increasing yearly, has reached 8% of the medical expenses in ophthalmology [18–20]. In Japan, bevacizumab is a relatively low-cost treatment, but it has not been approved for intravitreal use. Thus, it is used only as an off-label treatment.

There is no doubt that anti-VEGF treatment is effective, but due to financial limitations, the anti-VEGF treatments have been combined with panretinal photocoagulation, focal photocoagulation, and triamcinolone acetonide (TA) injections. Most ophthalmologists use anti-VEGF treatment as the first-line therapy and prefer the 1 + *prorenata*(PRN) regimen [21]. In the general clinical practice in Japan, the mean number of anti-VEGF injections was about 3 times a year, and ophthalmologists have tried to improve the therapeutic effects with a fewer number of anti-VEGF injections [22, 23].

Retinal experts in Japan have suggested that it is important to establish a multimodal approach to the treatment and management of DME. These multimodal treatments have been centered on the use of anti-VEGF treatment combined with TA injection, laser photocoagulation, or vitrectomy [24]. The results suggested that a TA injection was an effective treatment for DME among these treatment options. In addition, an earlier study has shown the effectiveness of TA injections in vitrectomized eyes in reducing the incidence of recurrences or persistence of the DME after a sub-Tenon triamcinolone acetonide (STTA) injection [25]. However, sustained-release steroid agents have not been approved in Japan. Therefore, intravitreal triamcinolone acetonide (IVTA) and STTA injections are being used in Japan.

Earlier studies have shown that in addition to VEGF, the levels of several inflammatory cytokines including interleukin-6 (IL-6), IL-8, interferon-induced protein-10 (IP-10), monocyte chemotactic protein (MCP-1), and intercellular adhesion molecule 1 (ICAM-1) are elevated in eyes with DME and RVO-ME. The inflammation has been found to be associated with the presence of the ME in DME and RVO [26–28]. Anti-VEGF agents can reduce the aqueous levels of VEGF, but they cannot reduce the levels of these other cytokines in eyes with DME. On the other hand, IVTA injections can significantly decrease the level of inflammatory cytokines including VEGF, IL-6, IP-10, and MCP-1 in eyes with DME [29].

Steroid treatments have been shown to be effective for the ME in eyes with a BRVO and CRVO [30, 31]. In eyes with DME, large-scale clinical trials have shown that steroid treatment can improve the degree of ME, and the improvements are comparable to that by anti-VEGF treatments in eyes with an implanted intraocular lens [32].

TA injections have a risk of cataract progression and intraocular pressure (IOP) elevation; thus, the first-line therapy for DME and RVO-ME is still intravitreal anti-VEGF injections. However, there may be benefits of switching from anti-VEGF to TA in eyes whose ME is refractory to the anti-VEGF agents. As best we know, there is no study examining the efficacy of switching from anti-VEGF to TA injections in eyes with DME or RVO-ME whose ME was refractory to anti-VEGF injections.

Thus, the purpose of this study was to determine the therapeutic effects and complications before and after switching from anti-VEGF injections to TA injections in eyes with DME or RVO-ME.

## 2. Patients and Methods

The medical records of 11 eyes of 11 patients with DME and 9 eyes of 9 patients with RVO-ME were reviewed. In the eyes with RVO-ME, 5 eyes had BRVO and 4 eyes had CRVO. All of the eyes had been treated with intravitreal anti-VEGF, and the ME was not resolved; i.e., ME was refractory to the anti-VEGF therapy. They were then switched to TA injections from April 2017 to July 2019.

Aflibercept was used for the anti-VEGF agent, and the treatment was performed with a regimen that consisted of an intravitreal injection at the time of diagnosis and readministered at the recurrence of a ME, i.e., the 1 + PRN regimen. The patients were examined every four weeks, and each examination included measurements of the central retinal thickness (CRT), best-corrected visual acuity (BCVA), and IOP.

### 2.1. Inclusion Criteria

Patients who met the following criteria were included. The anti-VEGF treatment had reduced the CRT to ≤300 *μ*m or by ≥200 *μ*m, but a recurrence of the ME had recurred at least 3 times over the same interval or within ±1 week. In addition, eyes that had received ≥6 anti-VEGF injections over a 32-week period and a recurrence still developed were included. For cases that met these two criteria, the anti-VEGF treatment was switched to TA injection at the time of the next recurrence.

The CRT, BCVA, IOP, and the mean interval for the recurrences were determined during the anti-VEGF treatment and after switching to the TA injections. The differences in the mean intervals of the recurrences during the intravitreal anti-VEGF injections and after switching to the TA injections were evaluated. The criterion that was used to determine that a recurrence of the ME had occurred was based on an increase of the CRT by ≥100 *μ*m both during the anti-VEGF treatment and after switching to TA injections.

The day of recurrence was set to be exactly midway between the visit when the recurrence of ME was observed and the previous visit when no recurrence was observed.

The CRTs, BCVAs, and IOPs were measured every 4 weeks before switching, immediately before switching (baseline), and every 4 weeks after switching to TA. The observation period was until the ME recurred twice after switching to the TA injection. If the time to the second recurrence was shorter than 6 months, the follow-up period was continued until 6 months due to the risk of an elevation of the IOP as a side effect of the TA injection. After a recurrence of ME, TA treatment was repeated if there were no side effects such as IOP elevation and the interval to recurrence was extended. The anti-VEGF treatment was performed if the interval was not extended. The BCVA was measured with a Landolt chart at every visit. The same protocol was used to measure the BCVA by five orthoptists for all patients.

The CRT was measured in the images obtained by spectral domain optical coherence tomography (SD-OCT; Heidelberg Engineering, Heidelberg, Germany). The macular thickness map program was used in obtaining the CRT for all patients. If the recurrence criteria were met again, retreatment with STTA, IVTA, or anti-VEGF agent was performed.

All BCVA measurements and OCT images were obtained by five orthoptists.

Also excluded were patients with steroid-induced ocular hypertension, primary or secondary glaucoma, active proliferative diabetic retinopathy, uveitis, and poor blood sugar control. Patients with other retinal diseases such as age-related macular degeneration, who had received another IVTA or STTA injection during the clinical course, and who had any intraocular operation within 12 months of the switching treatment were also excluded.

In all cases, focal and panretinal photocoagulation was performed before or within a few weeks of the initial anti-VEGF treatment. Cases that received photocoagulation within 12 months of the switching treatment were excluded. No photocoagulation was not performed during any other period. Patients with a history of systemic steroid use and vitrectomy in the study eye were also excluded.

For eyes with DME, earlier studies have reported that IVTA was more effective in improving the ME than STTA [33, 34]. However, IVTA has a higher risk of cataract formation than STTA. An earlier study of Japanese patients showed that cataracts developed in 2.04% of the eyes after IVTA and 1.55% after STTA [35]. Thus, we selected mostly STTA for phakic eyes and IVTA for pseudophakic eyes.

All patients had intravitreal injections of aflibercept (IVA), IVTA, or STTA by a standard method without complications. For IVA, 2 mg of aflibercept was injected. For IVTA, 4 mg of TA was injected, and for STTA, 20 mg of TA was injected. Preservative-free TA was used in all cases. Topical antibiotics were used for 3 days before and after the injections. When a significant elevation of the IOP to ≥22 mmHg was observed at the first visit after the TA injection, topical antiglaucoma medications were prescribed.

The clinical data and demographics of the patients before switching to the TA treatment are shown in Table 1. There were no significant differences in the sex distribution, BCVAs, CRTs, distribution of STTA and IVTA injections, phakic or pseudophakic, number of ME types (serous retinal detachment, cystoid macular edema, and sponge-like edema), and history of retinal photocoagulation. However, the RVO-ME group was significantly older than the DME group. The number of anti-VEGF treatments before switching to TA injection was greater in the RVO-ME group than in the DME group, but the difference was not significant.

All the procedures conformed to the tenets of the World Medical Association Declaration of Helsinki. Written informed consent was obtained from all patients, and approval for this study was obtained from the Institutional Review Board of the Chiba University Graduate School of Medicine (IRB#3541).

### 2.2. Statistical Analyses

The data are expressed as the means ± standarddeviations(SDs). For statistical analysis, the decimal visual acuities measured with a Landolt chart were converted to the logarithm of the minimum angle of resolution (logMAR). The significance of the differences in the findings was determined by Mann-Whitney *U* tests, Fisher's exact tests, chi-squared tests, Wilcoxon rank sum tests, or log-rank tests. A *P* < 0.05 was considered significant.

## 3. Results

### 3.1. Initial Response of CRT and BCVA after Switching to TA

**Figure 1:**
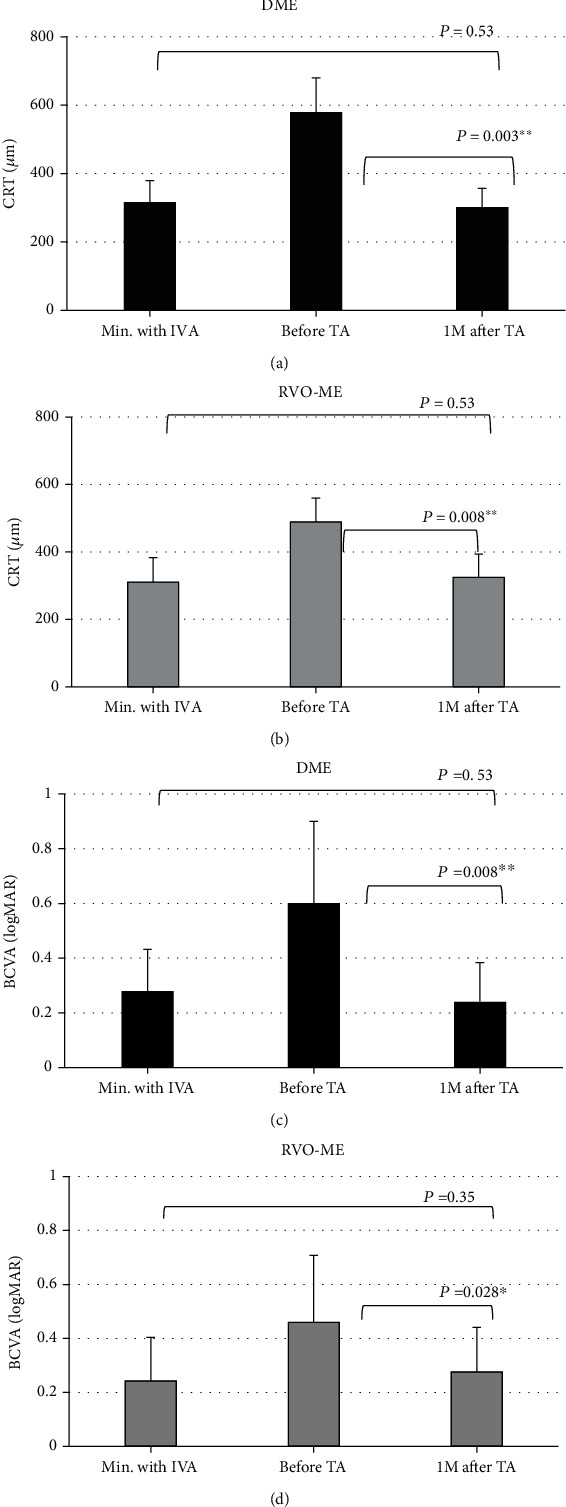
Comparisons of the central retinal thickness (CRT) under different treatment agents. The minimum CRT values during the anti-VEGF treatment, immediately before switching to triamcinolone acetonide (TA), and one month after the TA treatment in eyes with (a) diabetic macular edema (DME) and with (b) retinal vein occlusion macular edema (RVO-ME) are shown. In eyes with DME, the TA treatment significantly reduced the CRT from 580 ± 100*μ*m to 308 ± 62*μ*m at one month after beginning the TA treatment. The CRT was not significantly different from the minimum value of 308 ± 63*μ*m during the anti-VEGF treatment. Similarly, in eyes with RVO, the TA treatment significantly reduced the CRT from 489 ± 71*μ*m to 324 ± 70*μ*m at one month after beginning the TA treatment. The CRT was not significantly different from the minimum value of 310 ± 72*μ*m during the anti-VEGF treatment. Comparisons of the BCVA under different conditions. The minimum value during the anti-VEGF treatment, immediately before switching to TA treatment, and one month after TA treatment in eyes with (c) DME and (d) RVO-ME. In eyes with DME, TA treatment significantly improved the BCVA from 0.60 ± 0.30 logMAR units (Snellen 20/80) to 0.24 ± 0.14 logMAR units (Snellen 20/35). One month after the TA treatment, the BCVA was not significantly different from the minimum value of 0.28 ± 0.15 logMAR units (Snellen 20/38) during the anti-VEGF treatment. Similarly, in eyes with RVO, TA treatment significantly improved the BCVA from 0.46 ± 0.25 logMAR units (Snellen 20/58) to 0.28 ± 0.16 logMAR units (Snellen 20/38). One month after TA treatment, the BCVA was not significantly different from the minimum value of 0.24 ± 0.16 logMAR units (Snellen 20/35) during the anti-VEGF treatment. CRT: central retinal thickness; BCVA: best-corrected visual acuity; DME: diabetic macular edema; RVO-ME: retinal vein occlusion macular edema; TA: triamcinolone acetonide; IVA: intravitreal aflibercept.

In eyes with DME, the minimum CRT during the anti-VEGF treatment was 316 ± 63*μ*m, and it was 580 ± 100*μ*m immediately before switching to the TA treatment. It was then significantly reduced to 302 ± 55*μ*m at 1 month after the TA treatment (Figure 1(a)). In eyes with RVO-ME, the minimum CRT during the anti-VEGF treatment was 310 ± 72*μ*m, and it was 489 ± 71*μ*m immediately before switching to the TA treatment. It was then reduced significantly to 324 ± 70*μ*m at 1 month after the TA treatment (Figure 1(b)). The CRT at the last visit, i.e., when the ME recurred after switching to the TA injection, was 543 ± 129*μ*m in eyes with DME and 484 ± 48*μ*m in eyes with RVO-ME. The CRT in eyes with DME and with RVO-ME was significantly reduced at 1 month after switching to the TA injection compared to that before the TA injection (*P* < 0.05 for all; Figures 1(a) and 1(b)). There was no significant difference in the minimum CRT during the anti-VEGF treatment and at 1 month after switching to TA in eyes with DME and RVO-ME.

In eyes with DME, the minimum BCVA during the anti-VEGF treatment was 0.28 ± 0.15 logMAR units (Snellen 20/38), and it was 0.60 ± 0.30 logMAR units (Snellen 20/80) immediately before switching to the TA injection. It was then 0.24 ± 0.14 logMAR units (Snellen 20/35) at 1 month after the TA injection (Figure 1(c)). In eyes with RVO-ME, the BCVA during the anti-VEGF treatment was 0.24 ± 0.16 logMAR units (Snellen 20/35), and it was 0.46 ± 0.25 logMAR units (Snellen 20/58) immediately before switching to TA injection. It was then 0.28 ± 0.16 logMAR units (Snellen 20/38) at 1 month after the TA injections (Figure 1(d)). The BCVA at the last visit when a ME recurred after switching to TA injection was 0.36 ± 0.17 logMAR units (Snellen 20/46) in eyes with DME and 0.32 ± 0.17 logMAR units (Snellen 20/42) in eyes with RVO-ME.

In eyes with DME and RVO-ME, the BCVAs significantly improved after switching to TA compared to that before the switch (*P* < 0.05 for all; Figures 1(c) and 1(d)). There was no significant difference in the minimum BCVA during the anti-VEGF treatment and 1 month after switching to the TA injections. For both the DME and RVO-ME eyes, the effects of switching to TA injection were comparable to the effects of the anti-VEGF treatment.

### 3.2. Intraocular Pressure (IOP)

**Figure 2:**
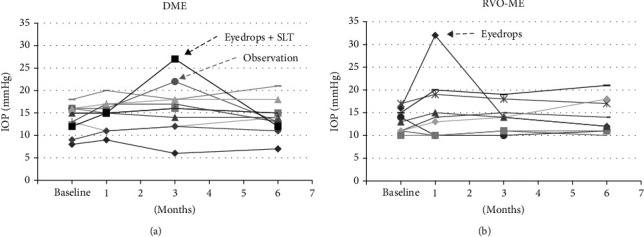
Changes of the intraocular pressure (IOP) before (baseline) and after switching to TA injections in eyes with DME (a) and RVO-ME (b). In eyes with DME, one eye required antiglaucoma eye drops and selective laser trabeculoplasty (SLT), and one eye had an increase in the IOP but then decreased without treatment. In eyes with RVO-ME, one eye required antiglaucoma eye drops. DME: diabetic macular edema; RVO-ME: macular edema associated with retinal vein occlusion.

An IOP elevation was observed in 2 of 11 DME eyes and 1 of 9 RVO eyes 3 months after switching to the TA injection. One eye from the DME group was not treated, and after 4 months, the IOP decreased to 13 mmHg. This eye received STTA injections. The IOP in one eye decreased with topical antiglaucoma medications, and in one eye, the IOP increased 3 months after beginning the TA injections and the IOP did not decrease with topical antiglaucoma medications but instead increased further. This eye then had selective laser trabeculoplasty (SLT) to reduce the IOP. Thereafter, no increase in the IOP was observed (Figures 2(a) and 2(b)). These two eyes received IVTA. The mean IOP at the last visit was 13.4 ± 3.5mmHg with a range of 7 to 21 mmHg in eyes with DME and 14.0 ± 3.6mmHg with a range of 10 to 21 mmHg in eyes with RVO-ME.

### 3.3. Mean Interval for Recurrence of ME

**Figure 3:**
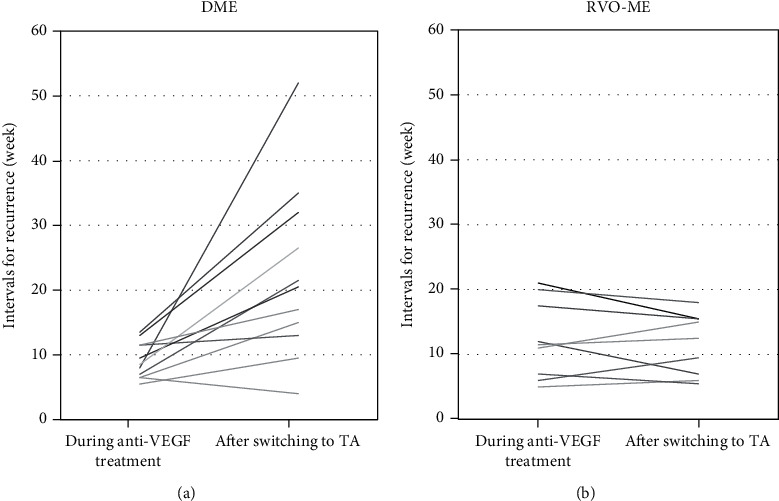
Changes of the intervals for recurrences during anti-VEGF treatments and after switching to TA treatment in eyes with DME (a) and RVO-ME (b). DME: diabetic macular edema; RVO-ME: retinal vein occlusion macular edema; TA: triamcinolone acetonide; VEGF: vascular endothelial growth factor.

**Figure 4:**
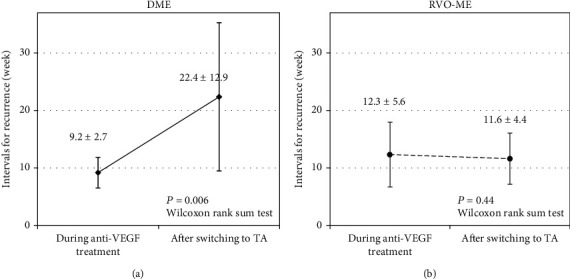
Changes in the mean intervals for recurrences during anti-VEGF treatment and after switching to TA treatment in eyes with DME (a) and RVO-ME (b). In eyes with DME, the mean intervals for recurrence were significantly extended from 9.2 ± 2.7 weeks to 22.4 ± 12.9 weeks after switching to TA treatment. In eyes with RVO-ME, the mean intervals for recurrence during anti-VEGF treatment and after switching to TA treatment were 12.3 ± 5.6 and 11.6 ± 4.4 weeks. There was no significant difference between these intervals.

Switching from anti-VEGF treatment to TA injections in eyes with DME extended the mean interval for recurrence to more than 8 weeks in 7 of 11 eyes and more than 16 weeks in 4 eyes. The extension of the mean interval for recurrences was 13.2 ± 12.2 weeks (range: -2.5-44 weeks; Figure 3(a)), and the mean interval for recurrence after switching to TA injections was significantly extended compared to that during the anti-VEGF treatment (*P* = 0.006; Figure 4(a)).

On the other hand, none of the eyes with RVO-ME had an extension of the mean interval for recurrence to more than 4 weeks after switching to the TA injection. The extension of the mean interval for recurrences was −0.72 ± 3.2 weeks (range: -5.5-4 weeks; Figure 3(b)). No significant extension was observed after switching to TA injection (*P* = 0.44; Figure 4(b)).

The extension of the mean interval for recurrence in DME eyes was 14.6 ± 13.6 weeks (range: 1.5-44 weeks) after STTA and 10.8 ± 8.7 weeks (range: -2.5-19 weeks) after IVTA. Although the number of cases was small, the difference in the mean intervals for recurrence between STTA and IVTA was not significant (*P* = 0.93).

### 3.4. Interval until Second Recurrence of ME

In the DME group, after the first recurrence, 6 of 11 eyes were treated with TA injection, and 5 of 11 eyes were treated with anti-VEGF treatment. The interval from the second treatment to a recurrence after switching to TA was defined as the second interval. In the DME eyes, the mean interval for recurrence during the anti-VEGF treatment before switching to TA was 9.3 ± 2.4 weeks (range: 6.5-13 weeks), and the mean interval for recurrence after switching to TA was 25.7 ± 13.2 weeks (range: 13-52 weeks). In these eyes, the mean second interval was 28.6 ± 9.4 weeks (range: 18.5-40.5 weeks). The difference between the mean first and second intervals for recurrence was not significant (*P* = 0.92). However, the difference between the mean interval for recurrence during anti-VEGF treatment before switching to TA and the mean second interval was significant (*P* = 0.027).

In DME eyes with anti-VEGF treatment as the second treatment, the mean interval for recurrence during anti-VEGF treatment before switching to TA was 9.1 ± 3.0 weeks (range: 5.5-13.5 weeks), and the mean interval for recurrence after switching to TA was 18.4 ± 11.2 weeks (range: 4-35 weeks). In these eyes, the mean second interval was 8.0 ± 4.8 weeks (range: 0-14.5 weeks). The difference between the first and second intervals for recurrence was not significant (*P* = 0.14), and the difference between the mean interval for recurrence during anti-VEGF treatment before switching to TA and the mean second interval was also not significant (*P* = 0.69).

In the RVO-ME group, treatment was returned to anti-VEGF treatment after switching to TA treatment and recurrences occurred in all cases. In one case, there was no response to anti-VEGF treatment. In other cases, the second interval to recurrence was not significantly different from the first interval and the interval for recurrence before switching to TA.

## 4. Discussion

The additional treatment was based on an increase in the CRT rather than a reduction of the BCVA. Many prior clinical studies have shown that the inverse correlation between the CRT and the BCVA is modest at best for eyes with either DME or RVO-ME treated by anti-VEGF agents and TA [30–32]. Thus, if the additional treatments were based on a reduction of the BCVA instead of an increase in the CRT, the results may have been different. However, Snellen chart-based BCVAs are used in the clinical practice in Japan. The accuracy and the reliability of Snellen charts are likely less than the ETDRS chart-based BCVAs. Because this was an observational study of patients examined in the clinic, the additional treatments were given by an increase in the CRT.

In Japan, most ophthalmologists use anti-VEGF treatment as the first-line therapy and prefer the 1 + *prorenata*(PRN) regimen [21]. All cases in this study were treated by anti-VEGF agents with the PRN regimen. The anti-VEGF treatment was sufficiently effective in all cases, but there was a clear recurrence at least 3 times over a specific interval. Even if the treatment is continued as it had been, there is a high probability of recurrences in the same interval.

All cases of DME and RVO-ME were treated by monthly anti-VEGF treatments from the early stage as in large-scale clinical trials or by the treat-and-extend regimen after monthly anti-VEGF treatments. However, multiple recurrences developed, and switching to TA injections may have been avoided. If not, it can make it difficult to generalize and compare the results of this study to other studies where monthly treatments were used.

As reported, the mean number of anti-VEGF injections was about 3 times/year in the general clinical practice in Japan [22, 23]. There may not be so many cases with multiple recurrences in a short interval, or it is possible that frequent anti-VEGF treatments have not been performed for such cases. In such a situation, the inclusion criteria in this study with at least 6 anti-VEGF injections over 32 weeks and a recurrence still developed may not be able to be directly applicable to most cases in Japan. However, there are several patients who have had such repeated recurrences, and some clinicians have difficulty in their medical options. We have focused on such patients with repeated ME recurrences.

The first-line therapy for eyes with DME or RVO-ME is intravitreal anti-VEGF injection. However, the results of earlier studies indicated that steroid treatment in eyes with DME can improve the BCVA, and the improvement was comparable to that after anti-VEGF treatment without the progression of cataracts [32]. There are also reports that steroids are effective in eyes with RVO-ME, but the anti-VEGF treatments are more effective [36].

Inflammatory cytokines other than VEGF have been reported to be involved in the development of the DME and RVO-ME. However, the level of the VEGF associated with the development of ME is different from that of other inflammatory cytokines. In DME, the levels of VEGF and inflammatory cytokines may be similar, but in RVO-ME, the level of VEGF is greater than that of the other inflammatory cytokines.

The anti-VEGF agents can reduce only the level of VEGF, but IVTA can reduce not only the levels of VEGF but also the other inflammatory cytokines [29]. In both the DME and RVO-ME eyes, it is believed that the VEGF levels are suppressed by repeated anti-VEGF injections but the ME recurs. Therefore, it has been suggested that the effects of inflammatory cytokines were higher in eyes with DME and the steroids should be more effective.

**Figure 5:**
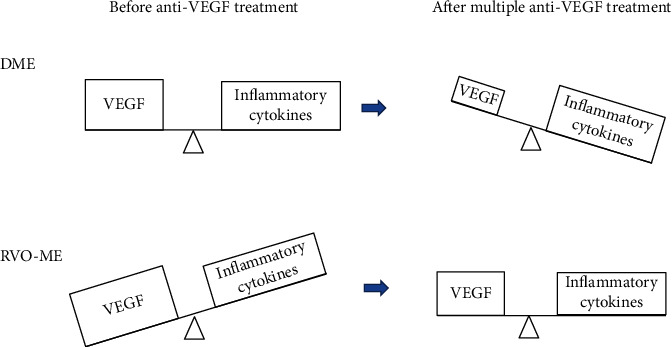
Schema comparing the magnitude and balance of the effect of VEGF and inflammatory cytokines on macular edema in eyes with DME and RVO-ME. VEGF: vascular endothelial growth factor.

On the other hand, the effects of inflammatory cytokines are weaker than VEGF in the early stages of RVO-ME, and the effects of inflammatory cytokines may not be so strong even if the VEGF levels are reduced by repeated anti-VEGF treatments (Figure 5).

Our subjects with DME and RVO-ME were refractory to anti-VEGF agents. However, we did include cases of anti-VEGF responders with multiple recurrences and excluded cases that did not respond to anti-VEGF agents at all. In the anti-VEGF nonresponders, the roles of the inflammatory cytokines other than VEGF may be different, and the response to TA in the eyes with RVO-ME might have been greater than shown. Thus, the findings in our cohort should be considered characteristic of cases with multiple recurrences, not as those of refractory cases including nonresponders. The term “suboptimal” response may be a more appropriate term than “refractory” response in describing the 20 eyes studied.

There were significant differences between the DME and RVO-ME groups in the baseline age and the prior photocoagulation treatments (Table 1). Thus, the RVO-ME group was significantly older than the DME group. However, the correlation between the age and the extension of the mean interval for recurrence was not significantly different for both groups (*r* = −0.086, *P* = 0.80 in the DME group; *r* = −0.50, *P* = 017 in the RVO-ME group; Spearman's rank correlation coefficient). On the other hand, panretinal photocoagulation (PRP) was performed in 10 of 11 eyes in the DME group and in 2 of 9 eyes in the RVO-ME group. The prior PRP may be associated with an extension of the mean interval for recurrence. In eyes with DME, the macular edema may be more likely to recur in eyes with prior PRP.

This study did not include eyes that had undergone retinal surgery such as vitrectomy but did include pseudophakic eyes. The DME group included 4 of 11 pseudophakic eyes, and the RVO-ME group included 3 of 9 pseudophakic eyes (*P* = 0.63). Thus, the effect of prior surgery was most likely not an important factor for the findings.

In eyes with RVO-ME, 5 had BRVO and 4 had CRVO, and they were combined in the statistical assessments because of the limited sample size. Our results showed that their responses to switching to TA were very similar. However, a stronger response to anti-VEGF and steroid therapy is generally expected in BRVO eyes than CRVO eyes. Thus, combining CRVO and BRVO into the same group might have confounded the results.

STTA and IVTA were used to administer the TA, and it has been reported that the effects of IVTA were greater in improving the ME than that of STTA in DME eyes [33, 34]. The ratio of STTA to IVTA for DME eyes was 7 : 4, whereas the ratio was 8 : 1 for RVO eyes (Table 1). Thus, the RVO eyes received STTA more often than IVTA relative to that in DME eyes. There was no significant difference in the distribution (*P* = 0.22), but it is possible that had more RVO eyes received IVTA, the interval for recurrences might have been longer.

Our results showed that there was no significant difference in the extension of the mean intervals for recurrence between IVTA and STTA. However, the small number of cases made the results of the statistical analyses weak.

An earlier study reported that the vitreous concentration of TA after an IVTA injection was much higher than that after an STTA injection. This difference could explain the earlier finding of a greater effect after IVTA [37]. The TA injected into the sub-Tenon space must pass through the sclera or through the general circulation to reach the vitreous cavity. Thus, its vitreous concentration must be significantly lower than that of TA, which may be the reason for the differences in the improvement of CRT and BCVA. However, the qualitative improvements of STTA and IVTA were the same, and the difference in the mean intervals for recurrence between STTA and IVTA was not significant.

In a meta-analysis study on the effects of STTA and IVTA on DME eyes, IVTA significantly improved the BCVA at 3 months. However, the benefit was not significant at 6 months, and the IOPs were significantly higher at three and six months [38]. A comparison of IVTA and STTA showed that IVTA significantly improved the BCVA and reduced the CRT at 3 months, but the improvement was not significant at 6 months and the IOPs were not significantly different [38, 39].

Both the IVTA and STTA treatments for DME have limited therapeutic effects. However, there were a certain number of cases of ME that were refractory to anti-VEGF treatments, and switching to TA treatments can often extend the mean interval for recurrences and may be considered a treatment option.

However, it has been reported that there was an IOP elevation (IOP > 21mmHg) in 14.7% of the 1406 eyes treated with STTA, and 7.5% required medication, and two eyes (0.14%) needed surgical procedures [40]. It is necessary to carefully consider the possibility and risk of IOP elevation when switching to TA treatments. For eyes with RVO-ME, switching to TA treatment for refractory ME had no merit and is not recommended due to the risk of IOP elevation.

Sustained-release steroid agents were not used because these agents have not been approved in Japan. Switching to these agents instead of IVTA or STTA may lead to longer-term control of DME and RVO-ME. However, it is necessary to pay more attention to the risk of IOP elevation because it has been reported that there was an IOP elevation (IOP > 25mmHg) in 29.7% of the 690 eyes treated with a dexamethasone intravitreal implant [41].

In Japan, the price of anti-VEGF agents is 17 times that of TA. In addition, this study showed that TA had the same therapeutic effects as anti-VEGF agents. In Japan, there are risks of IOP elevation and cataract progression, but frequent TA treatments are still more cost-effective than frequent anti-VEGF treatments. The therapeutic strategy of switching to TA is still a viable option as long as the clinicians and the patients are willing to accept the potential risk. Considering the potential financial burden, the treatment costs, and, above all, the loss of visual function in the event of glaucoma due to TA treatment, such a treatment strategy cannot be recommended in the case of RVO-ME. In contrast, in cases of DME, if switching to TA can significantly extend the interval to recurrences, it may be considered a treatment option.

There are limitations in this study other than the precautions and deficiencies as mentioned. First, this was a retrospective study with a small number of patients. And second, the lack of a control group for both DME and RVO-ME limits the conclusions that can be made. Thus, further large prospective studies are required to evaluate the efficacy and safety of IVTA and STTA for the treatment of refractory DME and RVO-ME after multiple anti-VEGF injections.

In conclusion, switching to TA treatments may be useful for the eyes with DME that are refractory to multiple anti-VEGF treatments. However, this option may be ineffective in eyes with RVO-ME. However, the risk of complications such as IOP elevations will be increased. We believe this study will provide useful information when ophthalmologists select the treatment for the management of refractory DME and RVO-ME in eyes with multiple anti-VEGF treatments.

## Figures and Tables

**Table 1:** Clinical data before switching to TA treatment.

	DME	RVO-ME	*P* value
Age (years), mean ± SD	60.0 ± 11.8	79.4 ± 8.0	0.002^∗^^1^
Men : women	7 : 4	5 : 4	0.53^∗^^2^
BCVA (logMAR), mean ± SD (Snellen equivalent)	0.60 ± 0.30 (20/80)	0.46 ± 0.25 (20/58)	0.28^∗^^1^
CRT (*μ*m), mean ± SD	580 ± 100	489 ± 71	0.08^∗^^1^
IOP (mmHg), mean ± SD	13.7 ± 2.96	13.0 ± 2.5	0.56^∗^^1^
STTA : IVTA	7 : 4	8 : 1	0.22^∗^^2^
Phakic eye : pseudophakic eye	7 : 4	6 : 3	0.63^∗^^2^
Type of macular edema	SRD: 3	SRD: 2	0.40^∗^^3^
	CME: 1	CME: 3	
	Sponge-like: 7	Sponge-like: 4	
History of photocoagulation treatment	PRP: 10	PRP: 2	0.006^∗^^3^
	Focal PC: 1	Focal PC: 3	
	No PC: 0	No PC: 4	
Number of anti-VEGF injections, mean ± SD (range)	13.1 ± 12.1 (6-50)	18.0 ± 9.4 (6-36)	0.07^∗^^1^
Duration of treatment with anti-VEGF (weeks), mean ± SD (range)	101 ± 71.2 (34-275)	231 ± 127 (32-417)	0.07^∗^^1^

DME = diabetic macular edema; RVO-ME = macular edema associated with retinal vein occlusion; SD = standard deviation; BCVA = best-corrected visual acuity; CRT = central retinal thickness; IOP = intraocular pressure; STTA = sub-Tenon triamcinolone acetonide injection; IVTA = intravitreal triamcinolone acetonide injection; SRD = serous retinal detachment; CME = cystoid macular edema; PRP = panretinal photocoagulation; PC = photocoagulation; VEGF = vascular endothelial growth factor.

## Data Availability

The data (Excel data that describes background and characteristics of the cases, and measured values such as BCVA, CRT, and IOP) used to support the findings of this study is available upon request from the corresponding author.
